# Transstadial Transmission of *Borrelia turcica* in *Hyalomma aegyptium* Ticks

**DOI:** 10.1371/journal.pone.0115520

**Published:** 2015-02-19

**Authors:** Zsuzsa Kalmár, Vasile Cozma, Hein Sprong, Setareh Jahfari, Gianluca D’Amico, Daniel I. Mărcuțan, Angela M. Ionică, Cristian Magdaş, David Modrý, Andrei D. Mihalca

**Affiliations:** 1 Department of Parasitology and Parasitic Diseases, University of Agricultural Sciences and Veterinary Medicine Cluj-Napoca, Cluj-Napoca, Romania; 2 National Institute of Public Health and Environment, Bilthoven, the Netherlands; 3 Department of Pathology and Parasitology, Faculty of Veterinary Medicine, University of Veterinary and Pharmaceutical Sciences, Brno, Czech Republic; 4 Biology Centre, Institute of Parasitology, Czech Academy of Sciences, České Budějovice, Czech Republic; University of Kentucky College of Medicine, UNITED STATES

## Abstract

*Borrelia turcica* comprises the third major group of arthropod-transmitted borreliae and is phylogenetically divergent from other *Borrelia* groups. The novel group of *Borrelia* was initially isolated from *Hyalomma aegyptium* ticks in Turkey and it was recently found in blood and multiple organs of tortoises exported from Jordan to Japan. However, the ecology of these spirochetes and their development in ticks or the vertebrate hosts were not investigated in detail; our aims were to isolate the pathogen and to evaluate the possibility of transstadial transmission of *Borrelia turcica* by *H. aegyptium* ticks. Ticks were collected from *Testudo graeca* tortoises during the summer of 2013 from southeastern Romania. Engorged nymphs were successfully molted to the adult stage. Alive *B. turcica* was isolated from molted ticks by using Barbour-Stoenner-Kelly (BSK) II medium. Four pure cultures of spirochetes were obtained and analyzed by PCR and sequencing. Sequence analysis of *glpQ*, *gyrB* and *flaB* revealed 98%–100% similarities with *B. turcica*. *H. aegyptium* ticks collected from *T. graeca* tortoises were able to pass the infection with *B. turcica* via transstadial route, suggesting its vectorial capacity.

## Introduction

Tick-borne infectious diseases are transmitted through the bite or ingestion of infected ticks [1]. Among the most widely distributed tick-borne bacterial pathogens are spirochetes of genus *Borrelia* [2, 3]. Genus *Borrelia* includes three main distinct phylogenetic lineages: Lyme borreliosis (LB) borreliae, relapsing fever (RF) borreliae and the recently described reptile-associated borreliae (REP) [4, 5]. The agents of LB are various species of the *Borrelia burgdorferi* sensu lato (s.l.) complex, all transmitted through the bite of hard ticks of genus *Ixodes* [6, 7]. RF *Borrelia* are transmitted by soft ticks, hard ticks or lice and have been reported primarily in northern US [8], Canada [9], Africa [10, 11] and Eurasia [12–16]. REP *Borrelia* isolates were described so far only from hard ticks or blood collected from tortoises.

In the last years, the epidemiologic role of reptiles has received increasing attention, mainly due to the international pet trade of animals originating from the wild [17]. Often, reptiles imported from the wild are harboring various tick species that facilitate the introduction of non-native tick-borne pathogens, thus posing a significant public health risk [17–19]. Various emerging and/or zoonotic pathogens have been isolated and characterized from reptiles or their associated ticks [20–25]. *Borrelia turcica*, a member of the REP group, was described from *H. aegyptium* ticks associated with Mediterranean tortoises in Turkey [21, 26]. *B. turcica* has been demonstrated to form a distinct monophyletic group and showed a relationship with both RF and LB groups. Based on the analysis of *flaB* and *gyrB* genes, respectively 16S rDNA sequences, *B. turcica* IST7 was distinct but branched off and clustered between the species of LB and RF *Borrelia* [21, 23, 26, 27]. Adeolu and Gupta (2014) grouped *B. turcica* spirochetes in a third distinct clade based on 16S rRNA gene [28]. REP *Borrelia* (*Borrelia* sp. tAG) spirochetes were also isolated from *Amblyomma geoemydae* ticks and they clustered with RF *Borrelia* based on another phylogenetic analysis [27].The natural cycle of tick-borne RF (TBRF) spirochetes involve a diversity of small mammals and their tick vectors [29]. They are a neglected cause of zoonotic diseases that result in significant illness and even death [30]. TBRF group of spirochetes currently includes ten already sequenced species of *Borrelia* [28], but many isolates are not yet characterized and further designations are expected.

Recently, *H. aegyptium* was shown to be an important carrier of various zoonotic agents: *Theileria* spp., *Rickettsia* spp. *Ehrlichia* spp., *Coxiella* spp., *Anaplasma* spp. and Crimean-Congo hemorrhagic fever virus [25, 31–35]. As nymphs and adults of *H. aegyptium* are found sporadically on humans they could play a role in the zoonotic transmission cycles of tick-borne pathogens [36–38]. Due to the low host specificity spectrum of hosts of *H. aegyptium* and its vectorial potential, these ticks could represent a threat to public health [37, 39, 40].

Considering the zoonotic potential of RF spirochetes, the increasing reptile trade and the common occurrence of reptile ticks on humans, studies aimed to elucidate the transmission patterns and vectorial competence of these ticks are needed. Except the tick-vertebrate cycle, the transstadial and transovarial transmission of *Borrelia* in ticks is also important for the maintenance of natural foci of pathogens [34]. Transstadial transmission of *Borrelia* spirochetes has been experimentally demonstrated for *B*. *burgdorferi* s.l. in various *Ixodes* species as well as in *Dermacentor variabilis* and *Amblyomma americanum* [41–43]. Additionally, transstadial transmission of REP *Borrelia* spp. (*Borrelia* sp. tAG) was demonstrated in *A*. *geoemydae* ticks [4, 27]. Nevertheless, there is no evidence about the possibility of transstadial transmission of *B. turcica* in ticks. The aim of the present study was to evaluate the possibility of transstadial passage of *B. turcica* in *H. aegyptium* ticks.

## Methods

### Collection of ticks and laboratory molting

In July 2013 six *T. graeca* tortoises (4 males, 2 females) were captured from Turcoaia locality in Tulcea County, Romania (45.09, 28.18). All of the tortoises were harboring ticks. Tortoises were brought to the laboratory of the University of Agricultural Sciences and Veterinary Medicine of Cluj-Napoca and kept for the duration of the natural tick feeding according to the national laws and ethical committee approvals. All ticks were allowed to feed until their full engorgement and spontaneous detachment. After detachment as well as after molting, all ticks were morphologically identified [44]. All detached nymphs were kept in the incubator at 21°C with 75% relative humidity and 12/12-hour light/dark photo period cycle at 7010 lux for four weeks to allow molting. The unengorged adults obtained as well as the still alive detached engorged adults were dissected for the isolation and collection of gut in order to allow the cultivation of spirochetes. After the tick detachment, all tortoises were released in their original habitat.

### Tortoises blood sampling

Blood was collected from each tortoise upon their arrival to the laboratory from the ventral caudal vein. The blood samples were collected on EDTA and stored at 4°C until PCR examination.

### Spirochete cultivation

Under a laminar flow cabinet adult ticks were dipped into 75% isopropyl alcohol for 5 min than air dried. Using forceps and scalpel blades, the midgut of each tick was dissected and inoculated individually into 7 ml of Barbour-Stoenner-Kelly (BSK) II medium (Sigma), incubated at 34°C and examined weekly for 2 months using dark-field microscopy (DFM) (Olympus BX53, magnification 200x). The BSK II medium contained 6% rabbit serum and was heat inactivated at 56°C 50 min prior to its use.

### Detection and identification of *Borrelia* species using PCR

DNA extraction was performed individually from each tick, each blood sample and each culture. DNA extraction was performed using a commercial DNA extraction kit (DNeasy Blood & Tissue Kit, Qiagen), according to the manufacturer’s protocols. The DNA quantity and purity were assessed using spectrophotometer analyses (NanoDrop Technologies model ND-1000, Inc., Wilmington, DE, USA).

*Borrelia* species were identified based on the region of 5S-23S rRNA (*rrf*-*rrl*) intergenic spacer (IGS), respectively on flagellin gene (*flaB*), glycerophosphodiester phosphodiesterase (*glpQ*) and DNA gyrase B subunit (*gyrB*) genes (Table 1). The PCR reaction was carried out in Bio-Rad T1000 Thermal Cycle and amplifications were performed according to previously described protocols [26, 45, 46]. Two additional set of primers were designed on the basis of the *glpQ* and *gyrB* gene sequences of *B. turcica* isolate IST7 (GeneBank accession no. AB529430, AB473535). The primer pairs (0.2 μM) were tested for optimal primer annealing temperature (ranging from 48 to 62) and were employed to detect with 100% spot matching two different *B. turcica* genes. The primers BTglpQF and BTglpqR (Table 1) were designed to target 910 bp long region of the *glpQ* gene of *B. turcica* and was amplified under the following conditions: 1 cycle of initial denaturation at 95°C 15 min followed by 40 cycles of denaturation at 94°C for 30 sec, annealing at 60°C for 1 min and extension at 70°C for 1 min with a final extension at 72°C for 10 min. To increase the sensitivity of the assay, BTgyrBF and BTgyrBR primers were used to detect DNA *gyrB* gene (1780 bp long region) of *B. turcica*. The amplification program comprised 15 min at 95°C followed by 40 cycles of denaturation at 94°C for 30 sec, annealing at 50°C for 1 min and extension at 70°C for 2 min with a final extension at 72°C for 15 min.

**Table 1:** Oligonucleotide primer pairs used.

Primer name	Sequence (5’…3’)	References
Rrf	CTGCGAGITCGCGGGAGA	[45]
Rrl	TCCTAGGCATTCACCATA	[45]
BflaA	TCTGATGATGCTGCTGGTATGG	[26]
BflaD	AGGTTTTCAATAGCATACTC	[26]
gyrB 3′	GGCTCTTGAAACAATAACAGACATCGC	[46]
gyrB 5′	GGTTTATGAGTTATGTTGCTAGTAATATTCAAGTGC	[46]
gyrB 5′+3	TTTATTGGTTTTAAGTCAAGTTGAATATGTC	[46]
BTgyrBF	GACCTGGTATGTATATTGGATCTG	In this study*
BTgyrBR	CTCTTCTAGGTTCAACATCATCTCCC	In this study*
glpQ-F	GGTATGCTTATTGGTCTTC	[54]
glpQ-R	TTGTATCCTCTTGTAATTG	[54]
BTglpQF	GCATTACCTCTAGTCATAGCTCATAGAGGTGC	In this study
BTglpQR	GCCTAATACTACACTAGGAAAATCTGTAAATACTCC	In this study

(* primers used in sequence analysis)

PCR reaction was carried out in a final volume of 25 μl using Ssofast EvaGreen Supermix (Bio-Rad). For each extraction procedure and PCR reaction, negative controls were used to check for cross-contamination of samples. Aliquots of each PCR product were electrophoresed on 2% agarose gel (1xTAE, pH 8.0) stained with SYBR Safe DNA gel stain (Invitrogen). The PCR products were purified by using QIAquick PCR purification kit (Qiagen) and further analyzed by sequence analysis (Macrogen Europe, Amsterdam). Nucleotide sequences were compared with those available in GenBank using Basic Local Alignment Search Tool.

### Phylogenetic analysis

Phylogenetic analysis was performed with Mega 6.06 software [47]. The evolutionary history was inferred using the Neighbor-Joining method [48]. The bootstrap consensus tree inferred from 1000 replicates is taken to represent the evolutionary history of the taxa analyzed. Branches corresponding to partitions reproduced in less than 50% bootstrap replicates are collapsed. The percentage of replicate trees in which the associated taxa clustered together in the bootstrap test (1000 replicates) are shown above the branches [49]. The evolutionary distances were computed using the Maximum Composite Likelihood method and are in the units of the number of base substitutions per site [50].

### Ethics Statement

The field study was carried out in Tulcea County, Romania (45.09, 28.18) based on research permits issued by the Research Authorization Department of the Danube Delta Biosphere Reserve Administration. The studies were not performed on private land, or on other location requiring specific permissions.

Veterinary conditions regarding protection of animals used in this research are complied according to national standards and legislation. The Research Bioethics Commission of University of Agricultural Sciences and Veterinary Medicine Cluj-Napoca (USAMV CN) committee reviewed and approved the document (Registration no. of approval of application: 10/2013). The Research Bioethics Commission of USAMV CN approved this study. This declaration certifies that the use of animals for research purpose within the experiment complies with the rules and regulations of the national (Law no. 206/2004 on good conduct in scientific research, technological development and innovation) and international (DIRECTIVE 2010/63/EU of the European Parliament on the protection of animals used for scientific purposes) legislation. No other ethics approvals were required, no animals were killed during the sample collection and ivasive methods were not used on the captured tortises.

## Results

### Ticks

**Fig 1:**
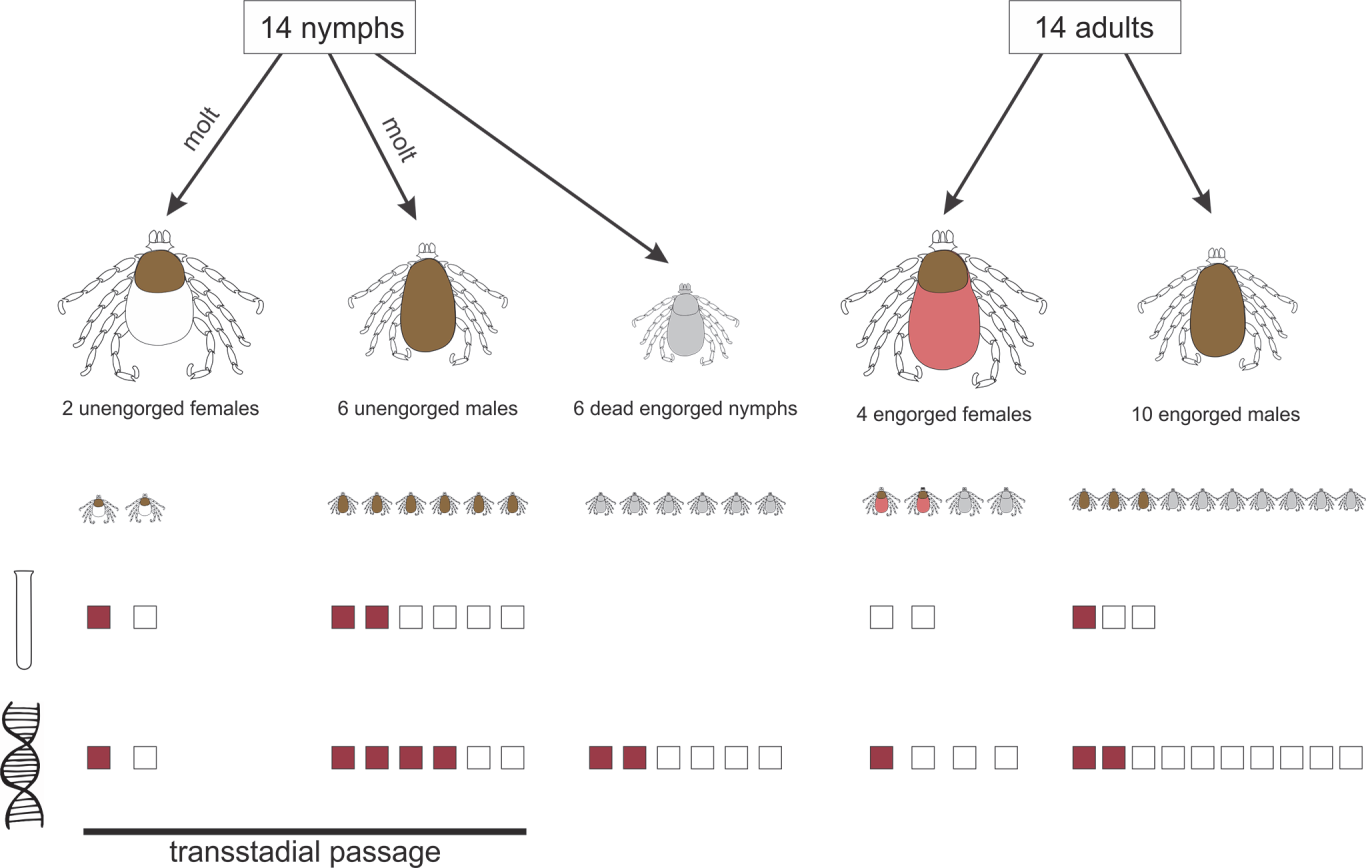
*H. aegyptium* ticks used in this study together with the PCR and cultivation results.

In total, 28 ticks were collected after full engorgement and spontaneous detachment from their tortoise hosts. After morphological examination, all were identified as *H. aegyptium*: 14 nymphs and 14 adults (4 females and 10 males). Out of the 14 fully engorged nymphs, 8 successfully molted after 3–4 weeks to the adult stage (resulting in 6 males and 2 females). Out of the 14 adults, only five (3 males and 2 females) were still found alive (Fig. 1).

### Spirochetes presence in cultures from the midgut of ticks

Cultivation of the intestinal content of the 8 unengorged adults and 5 alive engorged adults produced cultures positive for helically shaped and very motile spirochetes in 4 cases after 28 days of cultivation. Three cultures originating from unengorged adults (2 males and 1 female) resulting after laboratory molting of nymphs were positive for spirochetes at DFM. One culture obtained from an engorged adult male midgut was also positive for spirochetes by DFM (Fig. 1).

### Identification of spirochetes by PCR and sequencing

**Fig 2:**
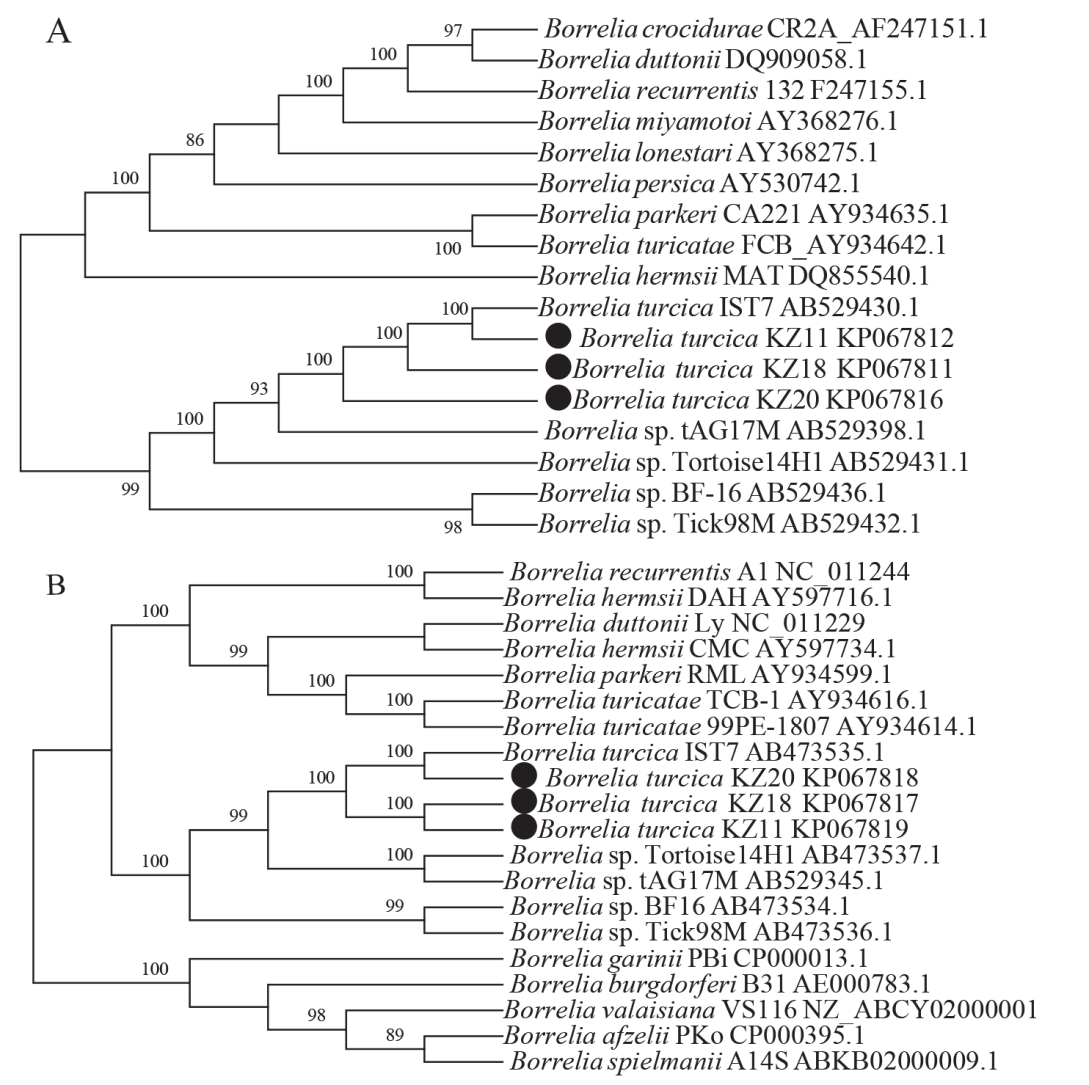
Phylogenetic analysis based on *glpQ* (A) and *gyrB* (B) genes of the genus *Borrelia*.

Ten *H. aegyptium* tick samples were positive for *Borrelia* using primers for *flaB*, *gyrB* and *glpQ* genes. All cultures were examined by PCR but only four (corresponding to those positive by DFM) were positive by using primers for *flaB*, *gyrB* and *glpQ* genes (Fig. 1). None of the 6 blood samples collected from *T. graeca* showed infection with *Borrelia* spirochetes by PCR, using the same primers. However, when using rrl/rrf primer pairs, a specific amplicon was not obtained by PCR of the IGS region. The PCR products were further analyzed by direct sequencing. Sequences were submitted to the GenBank sequence database (accession numbers: KP067811, KP067812, KP067816 for *glpQ* gene and KP067817, KP067818, KP067819 for *gyrB* gene). The *gyrB* and *glpQ* sequences showed 98%–100% similarities with reptile-associated *B. turcica* IST7. Phylogenetic analysis based on DNA sequences of *glpQ* and *gyrB* genes suggested that the sequences are distinct from LD, RF *Borrelia* and are similar with *B. turcica* IST7 and form together a monophyletic group (Fig. 2).

## Discussion

*H. aegyptium* is generally found in the Western Palearctic and in central and southwest region of the Asian continent [51]. These ticks are associated with tortoises and certain micromammals (e.g. rodents, hedgehogs) as well as humans and might play an important role in the tick-borne pathogen transmission [39]. In Romania, *T. graeca* and *Erinaceus roumanicus* (*E*. *roumanicus*) were reported to be hosts for *H. aegyptium* [52]. Numerous studies have been published about the carrier or vectorial capacity of *H. aegyptium* [25, 32]. In our study, *B. turcica* was successfully isolated from unengorged adult ticks (obtained by laboratory molting of naturally engorged nymphs) gut. These findings suggest that the collected nymphs were infected with *B. turcica* and transstadial transmission occurred from nymph to adult stage. This proof represents an important prerequisite for the vectorial capacity of *H. aegyptium* for *B. turcica*. For the definitive proof that *H. aegyptium* is a competent vector for *B. turcica*, further investigations are needed to prove the ability of the larva to nymph transstadial passage, transovarial transmission and tick to vertebrate transmission. However, we were not able to obtain eggs from engorged female ticks; hence transovarial transmission of *B. turcica* couldn’t be evaluated.

As all blood samples collected from tortoises were negative for *B. turcica*, the infection of nymphs might have occurred during a previous blood meal on another host, via transovarial transmission or by co-feeding mechanisms on either host. The absence of *B. turcica* in the blood of tortoises from this study as well as in some previous assays by us (Z. Kalmár, unpublished) and the relatively high prevalence in ticks collected from the same tortoises (data from this study as well as [53]) indicate the possibility that the natural vertebrate hosts for *B. turcica* could be micromammalian hosts (e.g. rodents, hedgehogs) which are preferred by immature stages. Nevertheless, negative *B. turcica* PCR can be associated with low bacteremia. However, Takano et al. [23] reported the absence of infectivity of REP *Borrelia* for mice following subcutaneous inoculation. Moreover, *B. turcica* was successfully isolated from different tissues (heart, skin, blood, muscle, urinary bladder) of tick-free wild *T. graeca* exported from Jordan to Japan, but not from intraperitoneally experimentally inoculated *Geochelone sulcata* tortoises [23]. Our results and the literature data suggest the possibility of more complex vector-pathogen-reservoir host interactions which need to be investigated in more detail. Additionally, the pathogenicity and zoonotic potential of *B. turcica* are not known.
